# Coherent selection of invisible high-order electromagnetic excitations

**DOI:** 10.1038/srep44488

**Published:** 2017-03-15

**Authors:** Ming Lun Tseng, Xu Fang, Vassili Savinov, Pin Chieh Wu, Jun-Yu Ou, Nikolay I. Zheludev, Din Ping Tsai

**Affiliations:** 1 Department of Physics, National Taiwan University, Taipei 10617, Taiwan; 2 Optoelectronics Research Centre and Centre for Photonic Metamaterials, University of Southampton, Southampton SO17 1BJ, UK; 3 Research Center for Applied Sciences, Academia Sinica, Taipei 115, Taiwan; 4 Centre for Disruptive Photonic Technologies, TPI, SPMS, Nanyang Technological University, Singapore 637371, Singapore

## Abstract

Far-field spectroscopy and mapping of electromagnetic near-field distribution are the two dominant tools for analysis and characterization of the electromagnetic response in nanophotonics. Despite the widespread use, these methods can fail at identifying weak electromagnetic excitations masked by stronger neighboring excitations. This is particularly problematic in ultrafast nanophotonics, including optical sensing, nonlinear optics and nanolasers, where the broad resonant modes can overlap to a significant degree. Here, using plasmonic metamaterials, we demonstrate that coherent spectroscopy can conveniently isolate and detect such hidden high-order photonic excitations. Our results establish that the coherent spectroscopy is a powerful new tool. It complements the conventional methods for analysis of the electromagnetic response, and provides a new route to designing and characterizing novel photonic devices and materials.

Nanophotonic systems such as photonic crystals^1^,^2^, metamaterials/metasurface^3^,^4^,^5^,^6^,^7^,^8^,^9^,^10^, plasmonic devices^11^,^12^,^13^,^14^,^15^,^16^, and dielectric resonators^17^,^18^,^19^,^20^, often rely on engineered electromagnetic resonances to achieve their unique functions. These resonances are commonly investigated and characterized by using two methods: far-field spectroscopy^10^,^21^,^22^ and mapping the near-field distribution^3^,^23^,^24^. The former can identify the wavelength of the resonance, and the latter can elucidate the type of the resonance. Although sufficient for relatively simple systems, both methods can fail in many complicated systems. Indeed, if several broadband resonances arise in the narrow wavelength range, the far-field spectrum will show complex features that can be too complicated to interpret. Furthermore, some weak resonances can be rendered invisible due to masking by a stronger resonance at a short wavelength separation. Such limitation of the conventional methods has hampered the development of advanced nanophotonic devices, and in particular ultrafast nanophotonic devices, where broad resonances are desired and spectral overlap may be unavoidable.

Here, by using three metamaterial samples, we demonstrate a new type of coherent absorption spectroscopy capable of solving this problem. Our method not only can disentangle complicated resonance features but can also detect hidden resonances, invisible in the conventional spectroscopy. This unique analytical power comes from our novel approach to electromagnetic excitation, which is provided by a standing wave formed by two coherent counter-propagating traveling waves (Fig. 1). We recently demonstrated the coherent absorption spectroscopy in experiment^25^,^26^,^27^ and theoretical simulation^28^,^29^. In this work, we go further in theoretical analysis, demonstrating the excitation of high-order resonances with particular symmetry and selective coupling to different multipole modes by using the spectroscopy.

High-order resonant modes are often excited simultaneously with the fundamental modes in nanophotonics^30^,^31^. Usually these modes are comparably weak and are thus often overlooked. However, they can become important both as a factor limiting the ultimate device performance, as well as a source of extra functionality, unavailable through use of low-order modes alone. Furthermore, the interference between the fields scattered by various high and low-order modes, can lead to a complicated spectrum. In the near-field, high-order modes are characterized by the unique field distribution that is useful for many purposes such as light trapping^12^,^32^ and sensing^22^,^33^,^34^. In this work, we demonstrate the detection and control of high-order resonance modes by using the coherent excitation. Our spectroscopic method, the coherent absorption spectroscopy, is important not only for correctly interpreting resonances, but also for controlling near-field related phenomena through selective multipole excitation, with applications in photovoltaics^35^, plasmon-enhanced fluorescence^36^,^37^ and nonlinear optical devices^38^,^39^,^40^.

## Results

### Detecting invisible hybridized dipole modes

We first discuss invisible modes induced by the hybridization of simple dipole resonances. Figure 2a is the schematic of the sample, an array of slit nanoantennas in a multi-layered thin film of Au/Si_3_N_4_/Au. The thicknesses of the middle Si_3_N_4_ layer and either Au layer are 50 nm and 30 nm, respectively. Each slit is 180 nm long and 40 nm wide. The periodicity of the array is 450 nm in both x and y directions. Figure 2b shows the numerically simulated spectra of the sample under traveling-wave illumination at normal incidence (i.e. the incident beam propagates in the z direction, see the Methods section for more details). Two strong absorption peaks are observed at 850 nm and 1500 nm.

We first use the conventional method (i.e. plotting the near-field distribution at peak wavelength) to interpret the two peaks. Figure 2c and d show the electric field and magnetic field, respectively, in the Au layer, at the peak wavelength of 850 nm. The field distributions can be readily associated with an electric dipole in the y direction and a magnetic dipole in the x direction. Very similar distributions are observed in the other Au layer, as well as in both layers at 1500 nm. The biggest difference between the metamaterial response at the two wavelengths is that, the instantaneous electric dipoles across the slits in the two Au layers are always parallel at 850 nm, whilst at 1500 nm they are always anti-parallel. Following the mode hybridization literature^41^,^42^, below we refer to the former (at 850 nm) as the symmetric mode, and to the latter (at 1500 nm) as the anti-symmetric mode.

**Figure 1:**
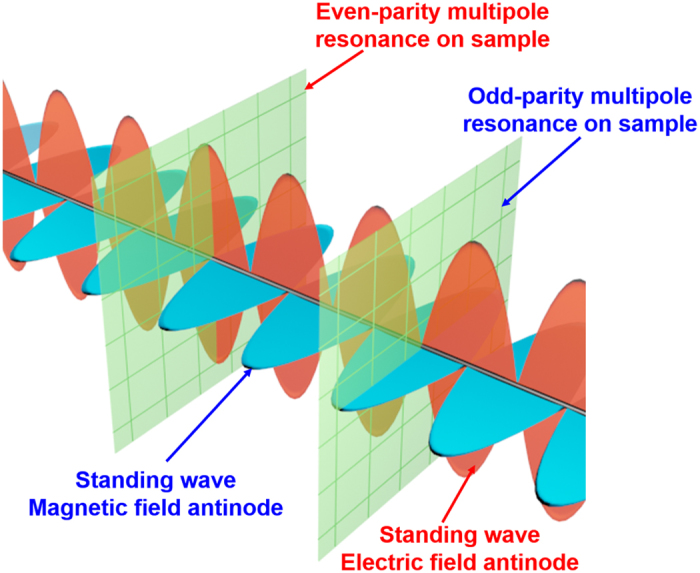
Coherent excitation of different resonance modes though selective coupling to odd and even parity multipoles. The coherent spectroscopy uses an optical standing wave as excitation. For a metamaterial at the electric antinode (E-antinode) of the standing wave, its symmetric modes are excited through coupling to even-parity multipoles. Meanwhile, for the same metamaterial at the magnetic antinode (B-antinode), its anti-symmetric modes are excited through coupling to odd-parity multipoles.

**Figure 2:**
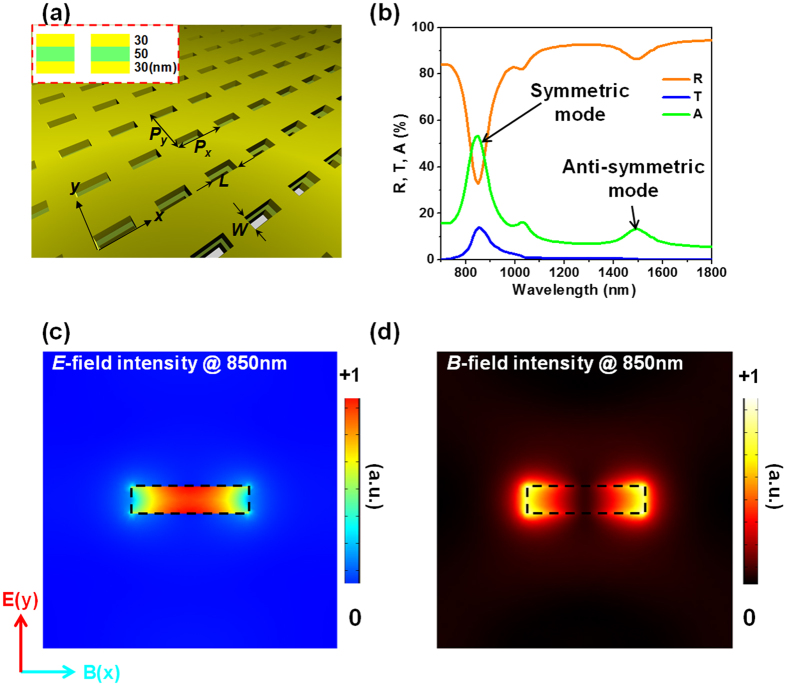
Fishnet metamaterial and the conventional analysis. (**a**) Schematic of the metamaterial. The metamaterial has two layers of Au (30 nm in thickness each, yellow colour) sandwiching a layer of Si_3_N_4_ (50 nm in thickness, green color). Unit cell dimensions: periodicity P_x_ = P_y_ = 450 nm, slit width W = 40 nm, slit length L = 180 nm. (**b**) Reflection R, transmission T, and absorption A spectra of the sample under traveling wave excitation. The symmetric and anti-symmetric modes are assigned in accordance with the conventional mode hybridization theory. (**c**) The electric field intensity in the middle plane of a Au layer. (**d**) The corresponding magnetic field intensity.

**Figure 3:**
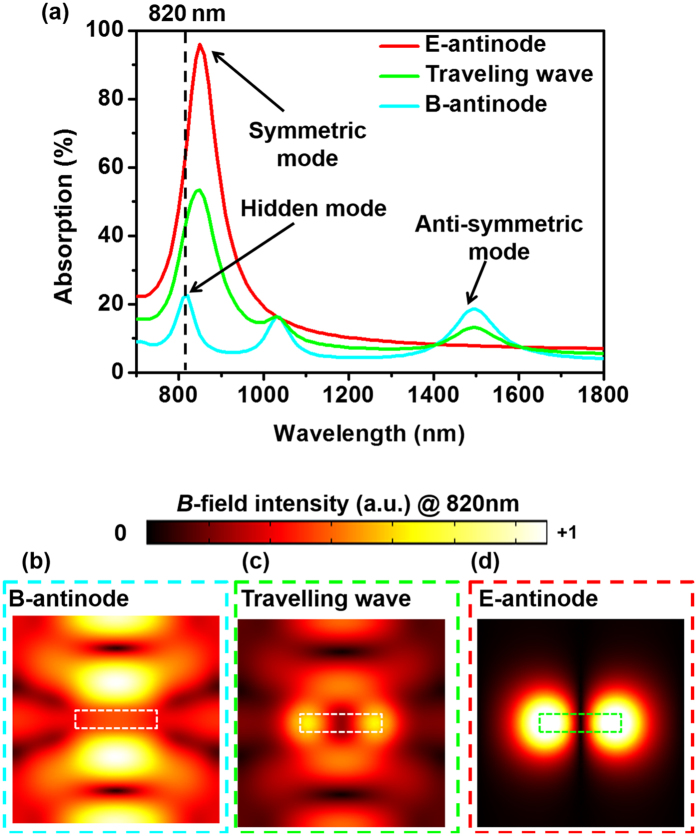
The fishnet metamaterial under coherent excitation. (**a**) Absorption spectrum of the sample under traveling-wave excitation, together with the two coherent absorption spectra with the sample at the E-antinode and the B-antinode of a standing wave. (**b–d**) The magnetic field intensity in the middle plane of the metamaterial under the three different excitation conditions. The excitation wavelength is 820 nm for all three cases.

We argue that, although the standard method of interpreting the electromagnetic response seems to fit very well with the results in Fig. 2, it is in fact insufficient. It overlooks a high-order resonance mode. This conclusion is supported by the two coherent absorption spectra in Fig. 3a. Firstly, before analyzing the results in Fig. 3, we discuss the behavior of symmetric and anti-symmetric metamaterial modes when placed into an optical standing wave. As shown in Fig. 1, the two coherent counter-propagating electromagnetic waves at the same frequency will create regions with vanishing magnetic field and strong oscillating electric field (E-antinodes), alternating with regions of strong oscillating magnetic field and vanishing electric field (B-antinodes). For a film at the E-antinode, the incident electric fields at its two surfaces always point in the same direction. Such excitation will couple strongly to even-parity multipoles, such as electric dipole, and therefore the electromagnetic energy will go into the symmetric metamaterial mode, which couples to free-space predominantly though the electric dipole. Meanwhile, the anti-symmetric modes, which couple to free-space via odd-parity multipoles (e.g. magnetic dipole and electric quadrupole), will be suppressed. Corresponding results can be drawn for the B-antinode, where the symmetric mode will be suppressed, and the anti-symmetric mode will be enhanced^43^.

We analyze the traveling-wave spectrum in Fig. 3 based on the following argument. One can see that the resonance observed at 1500 nm is a pure anti-symmetric mode: It is enhanced at the B-antinode and suppressed at the E-antinode. By contrast, the resonant response at 850 nm corresponds to an absorption peak in both E-antinode and B-antinode spectra. This implies that this peak, centered at 850 nm in traveling-wave absorption spectrum, is not purely symmetric or anti-symmetric. The symmetric mode centers at 850 nm and is dominant. Meanwhile, the anti-symmetric mode centers at 820 nm and is weak and effectively invisible. We stress that the resonance at 820 nm is effectively an invisible resonance in the conventional spectroscopy, as it cannot be reliably detected in the traveling-wave absorption, reflection, or transmission spectra (Fig. 2b).

To further demonstrate that the hidden resonance at 820 nm can be easily overlooked in the conventional method, we plot in Fig. 3b–d the near-field distribution for the three different excitation conditions. Very different field distributions are observed at the two coherent excitations (Fig. 3b and d): Near-field hot spots appear between adjacent slits at the B-antinode, while they appear at the two ends of each slit at the E-antinode. The field distribution for the traveling-wave excitation (Fig. 3c) shows a mixed feature: Magnetic hot spots appear both between adjacent slits and at the ends of each slit. This distribution is not significantly different from that at 850 nm (Fig. 2d), as the strongest local field is still at the ends of each slit. So we argue that, it is very difficult to notice the hidden resonance only from analyzing the near-field distribution. In comparison, the coherent spectroscopy is a very powerful tool to unveil such, otherwise invisible, resonance modes.

**Figure 4:**
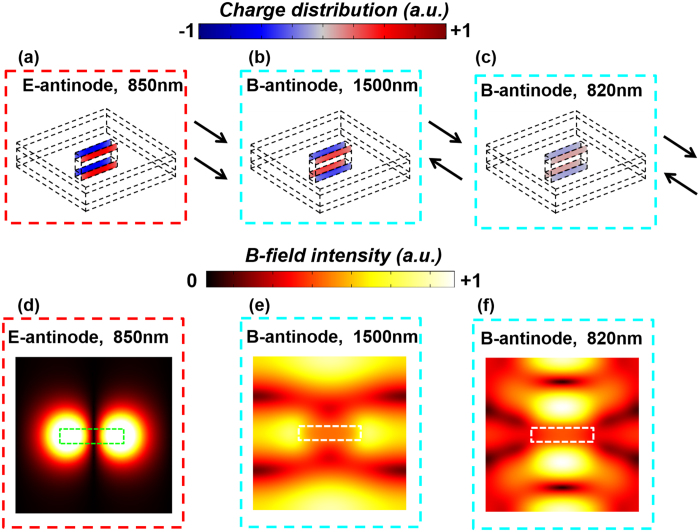
Near-field distribution of the fishnet metamaterial under coherent excitation. (**a–c**) The charge distribution inside the slits in the two Au layers under different excitation conditions at different wavelengths. (**b–d**) The corresponding magnetic field intensity in the middle plane of the metamaterial.

The invisible mode at 820 nm is in fact a high-order mode induced by the mode hybridization, as opposed to the two low-order modes centered at 850 nm and 1500 nm. All three modes are presented in Fig. 4, along with the corresponding near-field distributions. Figure 4a clearly shows that the resonance at 850 nm at the E-antinode is a pure symmetric mode: The charge distributions in the two Au layers are identical. Similarly, Fig. 4b shows that the resonance at 1500 nm at the B-antinode is a pure anti-symmetric mode: The charge distributions in the two Au layers have the same magnitude and opposite sign. Finally, from Fig. 4c we conclude that the mode at 820 nm is also an anti-symmetric mode. However, despite the similar charge distribution in the two anti-symmetric modes, the resonance at 820 nm shows a very different and more complicated magnetic field distribution, compared to that at 1500 nm (Fig. 4e and f). This indicates that the hidden resonance at 820 nm is a high-order resonance mode.

### Detecting invisible EIT resonance modes

**Figure 5:**
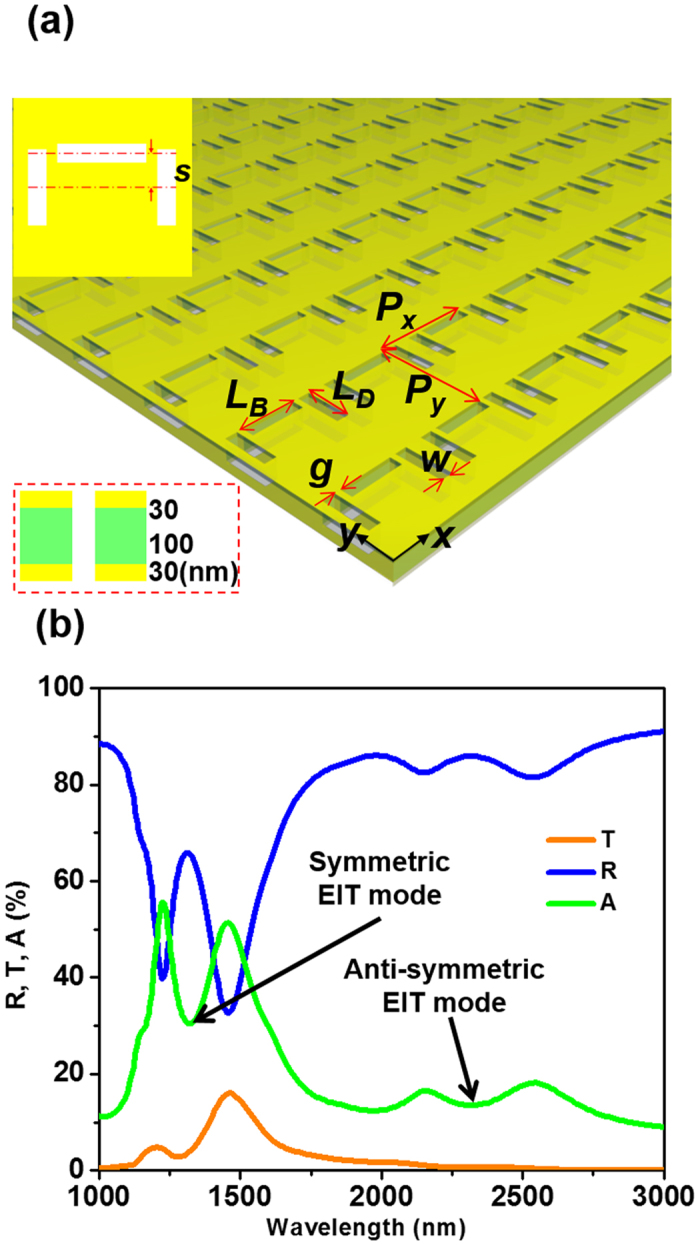
EIT metamaterial and its traveling-wave spectra. (**a**) Schematic of the metamaterial. Unit cell dimensions: periodicity P_x_ = P_y_ = 800 nm, slit width W = 90 nm, slit L_B_ = 400 nm, L_D_ = 340 nm, and gap g = 45 nm. (**b**) Traveling-wave spectra of the sample. The symmetric and anti-symmetric EIT modes are assigned based on the conventional mode hybridization theory.

We further discuss invisible high-order modes in a sample with more complicated near-field coupling. Figure 5a shows the schematic of the sample. Similar to the first sample, it is also a multi-layered thin film of Au/Si_3_N_4_/Au. Each unit cell contains three carefully arranged nanoslits, forming a shape that is commonly referred to as a dolmen metamolecule. In each Au layer, the near-field coupling between the slits generates resonances that resembles electromagnetically induced transparency (EIT) resonances^22^. Reference 44 further points out that, two EIT modes separated by a small distance will hybridize and form symmetric and anti-symmetric EIT modes. In similarity with most coupled oscillators, the symmetric mode will occur at higher resonant frequency and is therefore associated with resonant features at 1310 nm, whilst the anti-symmetric mode will occur at lower resonant frequency and is thus linked with resonant features at 2310 nm (Fig. 5b).

**Figure 6:**
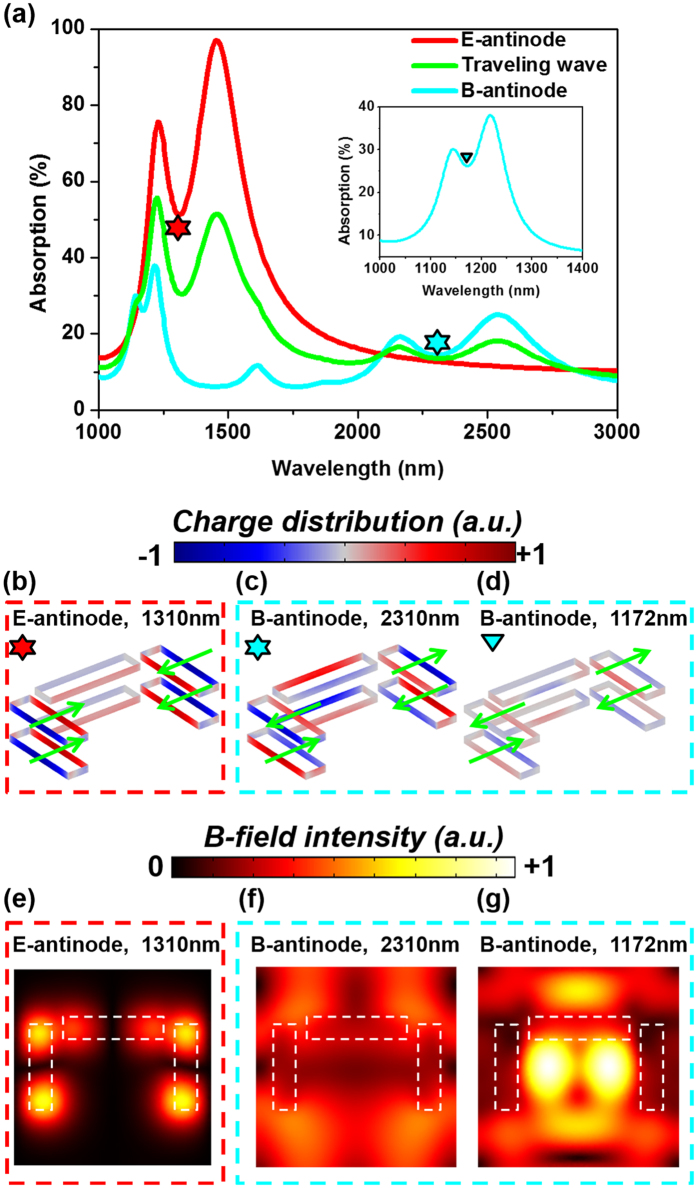
The EIT metamaterial under coherent excitation. (**a**) Absorption spectra of the sample under three different excitation conditions. (**b–d**) The charge density inside the slits in the two Au layers at different excitation conditions (E-antinode/B-antinode) and wavelengths. (**e–g**) Corresponding magnetic field distribution at the middle plane of the metamaterial.

However, we again argue that this hybridization model, in its present form, risks oversimplifying the response of the sample. Similar to the first sample, there is a hidden high-order resonance masked by low-order resonances in the traveling wave spectra. It can be readily recognized in the coherent spectra in Fig. 6a. The resonance at 2310 nm (wavelength defined as the dip in absorption) is a pure anti-symmetric mode: it is enhanced at the B-antinode and suppressed at the E-antinode. Very differently, the resonance centered at 1310 nm in the traveling-wave spectrum is not purely symmetric or anti-symmetric. The symmetric mode is centered at 1310 nm and is dominant. However, there is a third weak anti-symmetric mode, centered at 1170 nm, which is effectively invisible in the travelling wave spectrum. We note in passing that both the symmetric and the anti-symmetric modes near 1.3 μm show distinct EIT features.

The invisible resonance is a high-order anti-symmetric EIT mode. This conclusion comes from the charge distributions (Fig. 6b–d) and the magnetic field intensity (Fig. 6e–g). The low-order symmetric EIT mode shows identical charge distribution in the two Au layers, and the distribution in each layer resembles the electric quadrupole resonance (Fig. 6b). In comparison, the low-order anti-symmetric EIT mode shows opposite charge distribution in the two Au layers (Fig. 6c). The hidden resonance shows similar charge distribution to the low-order anti-symmetric mode, albeit with a much smaller amplitude (Fig. 6d). As before (in case of the first sample shown in Fig. 2), we observe similarity between the charge density distributions for the two anti-symmetric modes, at 1170 nm and 2310 nm (Fig. 6c,d), despite the stark difference between the corresponding maps of magnetic field (Fig. 6f,g). More details of the properties of this resonance mode can be found in the Supporting Information.

Very importantly, the hidden high-order resonance is a high-quality resonance. It has a significantly smaller full width at half maximum (FWHM) and higher quality factor compared to the two low-order resonances (Table 1). This result reveals an important application of the coherent spectroscopy: It can highlight and isolate high-quality resonances of a complicated system. This capability will be very beneficial to designing novel optical sensors^30^, nonlinear metamaterial devices^38^,^39^,^45^, and nanolasers^46^.

### Isolating hidden toroidal mode

**Figure 7:**
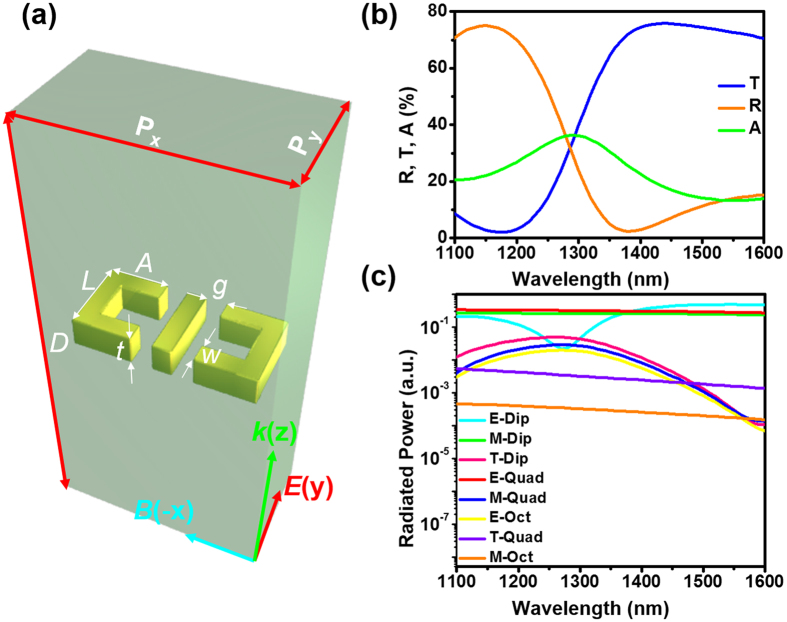
Toroidal metamaterial and its properties under traveling-wave excitation. Schematic of the metamaterial. Unit cell dimensions: periodicity in the x direction P_x_ = 220 nm, that in the y direction P_y_ = 120 nm, thickness of the dielectric cavity D = 500 nm, bar base length L = 80 nm, bar arm length A = 60 nm, bar width W = 20 nm, bar thickness t = 30 nm, gap g = 20 nm. (**b**) Traveling-wave spectra of the sample. (**c**) The contribution of different multipoles in radiation, including the electric dipole (E-Dip), magnetic dipole (M-Dip), toroidal dipole (T-Dip), electric quadrupole (E-Quad), magnetic quadrupole (M-Quad), toroidal quadrupole (T-Quad), electric octupole (E-Oct), and magnetic octupole (M-Oct).

In the third sample (Fig. 7), we not only show the detection of a high-order resonance mode but also identify its particular multipole type. Previously, we analyzed the behavior of the electric dipole, magnetic dipole, and electric quadrupole in a standing wave^25^. Here, based on our discussion for the first sample, we expand the analysis to include multipoles of even higher orders.

It can be shown that all electromagnetic multipoles emit fields of definite parity. It has also been shown that an infinite 2D array of identical oscillating multipoles, with sub-wavelength spacing, will emit radiation in plane waves perpendicular to the plane of the array^47^. Considering the radiation from such an array with just one kind of multipoles in each unit cell, one concludes that waves emitted by such array in front and back directions will either be identical, in case of even parity multipoles, or have a relative 180 degrees phase shift, in case of odd-parity multipoles. By reciprocity theorem^43^, the same applies in the opposite direction. An array of even-parity multipoles, such as electric dipoles, magnetic quadrupoles, toroidal dipoles, electric octupoles etc., will be effectively driven by two in-phase waves incident from opposite directions, i.e. it will be strongly excited when placed into the E-antinode. Correspondingly, the odd-parity multipoles, such as magnetic dipoles, electric quadrupoles, magnetic octupoles, toroidal dipoles, etc., will be strongly excited in the B-antinode.

Figure 7 is the schematic of the third sample. The metamaterial is an array of Au nanostructures buried in a Si_3_N_4_ dielectric cavity. The dimensions of the nanostructures are designed to show strong toroidal dipole resonance. The toroidal resonance has very unique near-field distributions of electric and magnetic fiels^48^,^49^,^50^. This property gives the resonance unique potential in light confinement, trapping and sensing. But it also renders the resonance very weak in natural materials and metamaterials, especially in the visible and near-infrared regimes^51^. For our sample, under the conventional traveling-wave excitation, the strength of the toroidal dipole is weaker than the magnetic dipole and electric quadrupole resonances in the whole calculated wavelength range (Fig. 7c).

**Figure 8:**
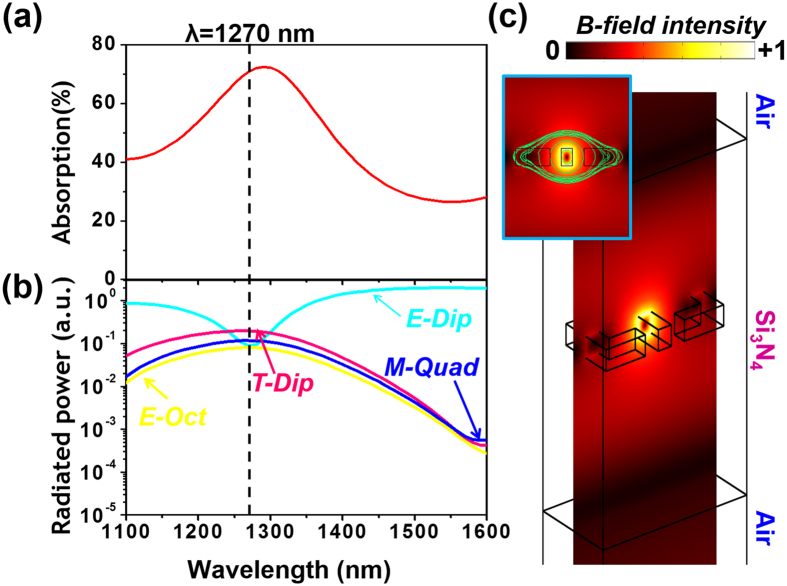
Isolating the toroidal dipole using coherent excitation. (**a**) The E-antinode absorption of the sample. (**b**) At this illumination condition, the contribution of different multipoles in radiation. (**c**) Corresponding magnetic field lines (green lines) and magnetic field intensity (color map) at the wavelength of 1270 nm, where the toroidal dipole dominates.

This weak toroidal resonance can be isolated by using the coherent excitation. Figure 8a shows the absorption of the sample at the E-antinode, and Fig. 8b shows the strength of different multipoles under this illumination condition. At the wavelength of 1270 nm, the toroidal dipole is the strongest scattering multipole excitation. This result can be understood from our analysis above. The magnetic dipole and electric quadrupole, the leading multipole terms under traveling-wave excitation at 1270 nm, emit odd-parity fields. They are suppressed at the E-antinode. Meanwhile, the toroidal dipole emits even-parity fields and is enhanced at the E-antinode. This difference makes toroidal dipole to the dominant multipole term at the E-antinode at 1270 nm.

Figure 8c further supports this conclusion with the near-field distribution of the metamaterial. A doughnut-shaped magnetic hot spot is observed, being the hallmark of a toroidal dipole resonance. The circulating magnetic fields have two contributions. (1) The Au nanostructures act as two asymmetrical split ring resonators in the xy plane, which generate anti-parallel magnetic fields in the z direction^3^. (2) The dielectric cavity supports displacement currents, which generate anti-parallel magnetic fields in the x direction^52^. Because the coherent spectroscopy can isolate and enhance the toroidal dipolar resonance, it is very useful for studying toroidal electrodynamics^51^ and developing photonic devices such as toroidal lasing spaser^53^.

## Discussion

In conclusion, we have demonstrated the detection of high-order resonances by using the coherent excitation. We have shown that high-order resonances of metamaterials are relatively weak, even totally invisible in the conventional spectroscopy. The coherent spectroscopy can identify and isolate such weak resonances, allowing for the unprecedented access to high quality resonances, and to selectively driving particular multipole excitations with free-space radiation. Based on the definite parity of all multipoles, we have discussed the behavior of high-order multipoles with the order up to the octupole. We also demonstrated that coherent spectroscopy can selectively target either odd- or even-parity multipoles. We have developed a planar toroidal metamaterial for the optical range and showed that its response can be addressed with multipole analysis despite the large thickness of the sample. The method we have developed can be used to analyze the electromagnetic properties of many different systems, including both metamaterials demonstrated here and natural materials that exhibit optical transitions other than the conventional electric dipole transition^54^,^55^,^56^. The spectroscopy provides very useful information of multipole resonances and transitions that complements the conventional traveling-wave spectra and near-field analysis.

## Methods

### Theoretical simulation

The resonant properties of metamaterials are obtained using the full-wave three-dimensional Maxwell equations solver (COMSOL Multiphysics) based on the finite element method. In the simulation, the periodic boundary condition is imposed along both the x and the y axis. The refractive index of Si_3_N_4_ is set as 2. The permittivity of Au is taken from the Drude-Lorentz model^57^ with a damping constant 0.140 eV and a plasma frequency 8.997 eV. The traveling-wave spectra can be readily obtained from the solver. In the calculation of the coherent absorption, the scattering boundary condition is applied to the top and the bottom surfaces of the simulated space, where two light waves are generated. By adjusting their relative phase difference, the central plane of the metamaterial films can be placed at the field antinode or node. The coherent absorption of metamaterial is then retrieved by comparing the total incident light intensity with the total scattered light intensity. For both the traveling-wave and the standing-wave excitations, the relative strength of the multipoles of the third metamaterial is calculated using the induced volume current density^58^.

### Data Availability

The data from this paper can be obtained from the University of Southampton ePrints research repository: http://dx.doi.org/10.5258/SOTON/D0010.

## Additional Information

**How to cite this article:** Tseng, M. L. *et al*. Coherent selection of invisible high-order electromagnetic excitations. *Sci. Rep.* **7**, 44488; doi: 10.1038/srep44488 (2017).

**Publisher's note:** Springer Nature remains neutral with regard to jurisdictional claims in published maps and institutional affiliations.

## Supplementary Material

Supplementary Information

## Figures and Tables

**Table 1:** Comparison of the quality of the three EIT resonances.

Resonance wavelength (nm)	FWHM (nm)	Quality factor
1310 (low-order visible mode)	130	10.2
1172 (high-order hidden mode)	39	30
2310 (low-order visible mode)	100	23.1
