# Supplementary material for: Coherent selection of invisible high-order electromagnetic excitations
Source: Sci Rep. 2017 Mar 15;7:44488. doi: 10.1038/srep44488 (PMC5353631; doi:10.1038/srep44488)
Supplement: Supplementary Information [file srep44488-s1.pdf]

Supplementary Information

Coherent selection of invisible high-order electromagnetic excitations

Ming Lun Tseng[1*], Xu Fang[2*], Vassili Savinov[2*], Pin Chieh Wu[1,3], Jun-Yu Ou[2], Nikolay I. Zheludev[2,4†], and Din Ping Tsai[1,3†]

[1]*Department of Physics, National Taiwan University, Taipei 10617, Taiwan*

[2]*Optoelectronics Research Centre and Centre for Photonic Metamaterials, University of Southampton, Southampton SO17 1BJ, UK*

[3]*Research Center for Applied Sciences, Academia Sinica, Taipei 115, Taiwan*

[4]*Centre for Disruptive Photonic Technologies, Nanyang Technological University, Singapore 637371, Singapore*

* *These authors provided equally important contribution to this work.*

† *Correspondence and requests for materials should be addressed to N.I.Z. or D.P.T. (email: niz@orc.soton.ac.uk & dptsai@phys.ntu.edu.tw).*

The second sample discussed in the main text shows a hidden EIT resonance. In this Supporting Information, we further discuss its dependence on the nanostructures, as well as unveil the near-field coupling that causes the resonance.

In nanophotonic systems, the EIT resonance can be induced by introducing structural asymmetry.[1-3] This is revealed in Figure S1, which shows a series of samples with different degrees of structural asymmetry. The asymmetry is characterized as s, the lateral displacement of the center slit with respect to the center of the unit cell. Figure S1a compares the B-antinode absorption spectra of these samples. For the structurally symmetric sample (i.e. $s = 0$), a single absorption peak is observed at 1166 nm. The EIT feature appears with introducing the structural asymmetry and becomes very obvious for $s = 155\ nm$ (the sample used in the main text) and $s = 215\ nm$.

For the sample with $s = 155\ nm$, its EIT resonance originates from the interference between a bright and a dark magnetic resonance, which is revealed in Figures S1b and Figure S1c. Figure S1b shows the distribution of the magnetic field polarized along the x axis. It is the bright mode, as it has the same polarization as the incident magnetic field and shows finite net intensity. In comparison, Figure S1c shows the magnetic field polarized along the y axis. It is the dark mode, as its direction is orthogonal to the incident magnetic field and it has no net total intensity. The hidden EIT resonance can

thus be attributed to the interference between the bright magnetic resonance (Figure S1b) and the dark magnetic resonance (Figure S1c).

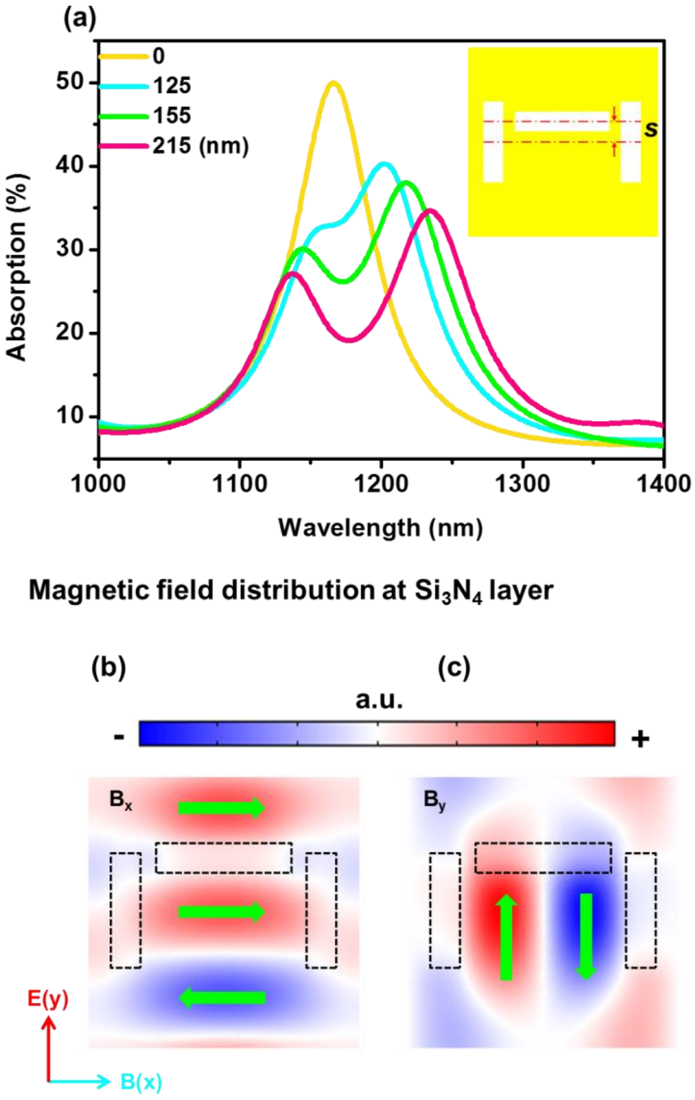

Figure S1. (a) B-antinode absorption spectra of four samples with different degrees of structural asymmetry. (b-c) Distribution of the magnetic field polarized along (b) the *x* axis and (c) the *y* axis for the sample with *s* =155 nm. The green arrows indicate field direction.

References for supporting information

1. Hao, F., Sonnefraud Y., Dorpe P. V., Maier S. A., Halas N. J., Nordlander P. Symmetry Breaking in Plasmonic Nanocavities: Subradiant LSPR Sensing and a Tunable Fano Resonance. *Nano Lett.* **8**, 3983-3988 (2008).

2. Liu, N., *et al.* Planar Metamaterial Analogue of Electromagnetically Induced Transparency for Plasmonic Sensing. *Nano Let.* **10**, 1103-1107 (2010).

3. Plum, E., Tanaka K., Chen W. T., Fedotov V. A., Tsai D. P., Zheludev N. I. A combinatorial approach to metamaterials discovery. *J. Opt.* **13**, 055102 (2011).
